# Readiness of ethics review systems for a changing public health landscape in the WHO African Region

**DOI:** 10.1186/s12910-015-0078-9

**Published:** 2015-12-02

**Authors:** Marion Motari, Martin Okechukwu Ota, Joses Muthuri Kirigia

**Affiliations:** World Health Organization, Regional Office for Africa, Brazzaville, Congo

**Keywords:** National ethics review committees, Ethics of health research, Research and development, African Region

## Abstract

**Background:**

The increasing emphasis on research, development and innovation for health in providing solutions to the high burden of diseases in the African Region has warranted a proliferation of studies including clinical trials. This changing public health landscape requires that countries develop adequate ethics review capacities to protect and minimize risks to study participants. Therefore, this study assessed the readiness of national ethics committees to respond to challenges posed by a globalized biomedical research system which is constantly challenged by new public health threats, rapid scientific and technological advancements affecting biomedical research and development, delivery and manufacture of vaccines and therapies, and health technology transfer.

**Methods:**

This is a descriptive study, which used a questionnaire structured to elicit information on the existence of relevant national legal frameworks, mechanisms for ethical review; as well as capacity requirements for national ethics committees. The questionnaire was available in English and French and was sent to 41 of the then 46 Member States of the WHO African Region, excluding the five Lusophone Member States. Information was gathered from senior officials in ministries of health, who by virtue of their offices were considered to have expert knowledge of research ethics review systems in their respective countries.

**Results:**

Thirty three of the 41 countries (80.5 %) responded. Thirty (90.9 %) of respondent countries had a national ethics review committee (NEC); 79 % of which were established by law. Twenty-five (83.3 %) NECs had secretarial and administrative support. Over 50 % of countries with NECs indicated a need for capacity strengthening through periodic training on international guidelines for health research (including clinical trials) ethics; and allocation of funds for administrative and secretariat support.

**Conclusions:**

Despite the existing training initiatives, the Region still experiences a shortage of professionals trained in health research ethics/ethicists. Committees continue to face various capacity needs especially for evaluating clinical trials, for monitoring ongoing research, database management and for accrediting institutional ethics committees. Given the growing number of clinical trials involving human participants in the African Region, there is urgent need for supporting countries without NECs to establish them; capacity strengthening where they exist; and creation of a regional network and joint ethical review mechanisms, whose membership would be open to all NECs of the Region.

## Background

All research on humans should be conducted in accordance with basic ethical principles of respect for persons, beneficence and justice. It is the duty of Ethics Review Committees (ERCs), whether national or institutional, to review and approve proposals that demonstrate this standard [1]. In 2001 the 51^st^ session of the WHO Regional Committee for Africa expressed concerns about some health-related studies undertaken in the Region which had not been subjected to any form of ethics review [2]. Following this, a rapid survey of national ethics review committees (NECs) was conducted in 2003 in 28 countries of the WHO African Region. The survey revealed that 36 % of these countries did not have ERCs, 15 % indicated that ethical approval of research proposals was not required [3].

A similar study conducted in 2008 revealed that regulatory infrastructures and independent oversight processes that could minimize the risk of exploitation were not-so-firmly established; NECs were inadequately supported financially and could not maintain and sustain themselves; they were faced with operational challenges stemming from a dearth of trained bioethicists/ethicists and/or adequately experienced committee members skilled enough to undertake ethics review [4, 5]. This raises concerns as to whether research ethics principles can be adequately enforced in these countries. There is need for identifying gaps and strengthening capacities of countries to respond to a globalized biomedical research system which is constantly challenged by new public health threats, rapid scientific and technological advancements affecting biomedical research and development (R&D), delivery and manufacture of vaccines and therapies.

Ministers of health of countries in WHO African Region endorsed the Algiers Declaration and committed themselves to, among others [6]:

- establish or strengthen governance structures to promote ethics and increase public trust in research;
- establish appropriate mechanisms for scientific and ethical oversight of research for health, including regulation of clinical trials and sensitization of the people to their role, their rights and their obligations in research for health;
- develop a critical mass of focal persons and well-trained national researchers, including those working abroad, in various disciplines and areas of health research, including ethics and regulation; and to
- establish norms and standards, including ethical ones, taking into account technological progress and new knowledge management methods.

This provides a rich backdrop for investigating how well-equipped national ethics committees in countries of the WHO African Region are to safeguard the dignity, rights and safety of research participants in the current public health landscape. This study was conducted in 2012 to assess the readiness of NECs to provide adequate ethical oversight on health research conducted in countries; and to identify areas for capacity strengthening. Readiness of NECs was assessed against their implementation of the following three inter-dependent themes described below.

- *Governance of health research ethics*

International guidelines such as the Declaration of Helsinki [7], the Council for International Organization of Medical Sciences (CIOMS) international ethical guidelines [1, 8], the International Conference on Harmonization of Good Clinical Practice (ICH-GCP) guidelines [9], and WHO’s standards and operational guidance for ethics review [10], provide ethical standards for conducting health research involving human participants. While these international guidelines provide a sound operational framework for governing ethical research, they are not enforceable in most countries of the African Region since they do not have the force of law. It therefore becomes necessary for countries to adopt these standards, taking into account their unique national health research needs and contexts, into a form that can be enforced by law. A legally binding framework, as has been argued by Chima [11] would provide a measure of protection for research participants.

b. *Existence of mechanisms for ethical review of health research*

National Ethics Committees have the responsibility for safeguarding the dignity, rights and safety of research participants in their respective countries. This responsibility is not only accomplished through the ethical review and approval of health research proposals. It is a responsibility with a longer life-span requiring NECs to continuously monitor and evaluate health research, through reports and site visits, to ensure compliance with ethical standards and with the approved research protocol [12].

c. *Continuous ethics training and capacity requirements*

To be able to perform its functions, a NEC needs to have the requisite ethics review competencies/capacities among its members and the necessary administrative and logistical support. The ethics review competencies can be acquired through formal training or through “learning by doing”. The Committee should therefore have some members formally trained in ethics, while ensuring a balanced representation of disciplines, interest groups and even gender. Continuous ethics training and capacity building opportunities should be availed to all members of the Committee to ensure that they stay abreast with new developments in the field. The importance of permanent and reliable secretarial, administrative and logistical support for the smooth functioning of a NEC cannot be gainsaid.

## Methods

The readiness and capacities of NECs to cope with the current global ethical review requirement was assessed by including questions addressing the three themes mentioned above. Table 1 below provides a summary of sample questions that were asked in the survey under each theme.

**Table 1:** Sample questions on survey questionnaire

Theme 1: Existence of national framework for governing ethical review
• Is there a law/policy establishing a national ethics committee?
• What are the main functions of the national ethics committee?
• Are there written guidelines for constituting (i.e. selecting members) the national ethics committee?
• What year was the first national ethics committee established?
• Is there a policy/law in your country that establishes the applicable ethical standards for research involving human subjects?
• Are regional or international guidelines referred to in the national laws?
• Are all health research institutions in your country required to adopt a policy regarding research ethics?
Theme 2: Existence of mechanisms for ethical review
• Does the national ethics committee have standard operating procedures to guide its work?
• If some members of the committee disagree with the majority’s decision, is the substance of the disagreement recorded and communicated?
• Is there an explicit mechanism for excluding committee members with a direct interest in a proposal for review from participating in its assessment?
• Are there penalties for non-compliance with the regulations and decisions of the national ethics committee?
- If so, what are the penalties?
• Are there mechanisms in place for monitoring ongoing research?
• What types of mechanisms are applied for monitoring ongoing research?
- Annual ethical review of previously approved projects
- Required periodic written report from principal investigator
- Unannounced audit of research by representatives of ethical review committee
- Other
Theme 3: Continuous ethics training and capacity requirements
• Is training/capacity building opportunities provided to members of the national ethics committee?
• Is this training required or optional for members of the ethics committee?
• Is the ethics committee familiar with the following: CIOMS guidelines, WHO guidelines, declaration of Helsinki, Nuremberg Code?
• Does the committee engage the services of consultants from time to time for technical support?
• Is there any secretarial and administrative support for the national ethics committee?
• What in your view are the specific capacity needs of the national ethics committee?
• What in your opinion could be done to improve the performance of the national ethics committee?

To assess governance of health research ethics, the survey questionnaire elicited information regarding the existence of law/policy for the creation of NECs, including a provision establishing ethical standards for research involving human participants, and whether reference is made to any regional or international guidelines for medical research. Other questions elicited information on clear provision for NEC membership criteria; composition and functions of NECs; and whether all research institutions were required to adopt policy regarding research ethics.

As concerns the existence of mechanisms for ethical review of health research proposals, the questionnaire elicited information on the existence of NECs with standard operating procedures (SOPs) including clear guidelines for decision-making and managing scenarios of conflict of interest. The questionnaire also elicited information on mechanisms for monitoring on-going research; and penalties for non-compliance with ethical standards. The questions for this section used open-ended questions such as “*Are there penalties for non-compliance with the regulations or decisions of the national ethics committee*?” and “*If so, what are the penalties*?” which allowed survey respondents to provide specific details of their respective country contexts. (More examples provided on Table 1 below).

To determine provision for continuous ethics training and capacity strengthening the questionnaire elicited information on training requirements for NEC members; and the availability of opportunities for capacity strengthening for committee members. An inquiry was made on whether NECs were familiar with a number of key international guidelines and declarations (Helsinki, CIOMS, WHO, Nuremberg code) as a proxy to assess the level of basic knowledge and awareness of international health research ethics by committee members. A number of questions on the availability of permanent and reliable secretarial, administrative and logistical capacities for the smooth-running of NECs were included in the questionnaire. In order to allow for key capacity needs to emerge from the survey, the following two-open ended questions were asked at the end of the questionnaire:

- *What in your view are the specific capacity needs of the national ethics committee?*
- *What in your opinion can be done to improve the performance of the national ethics committee?*

The survey questionnaire was developed in English and was externally reviewed by experts in the field to ensure relevance and coherence. It was then translated into French and was sent to ministries of health through the offices of WHO Representatives in the 20 Anglophone and 21 Francophone countries respectively. The questionnaire was not translated into Portuguese and was therefore not administered to the 5 Lusophone countries in the Region. The targeted respondents were senior officials in the ministry of health, who by virtue of their offices were considered to have expert knowledge of the research ethics review system in their respective countries.

### Limitations of the survey

The questionnaire was sent to 41 out of the 47 countries of the WHO African Region. The five Portuguese speaking countries were omitted, and thus the results might not be generalizable to Portuguese speaking countries. South Sudan was also not included in the survey since it was not among Member States of the African Region at the time.

Secondly, the types of questions asked by this survey mainly focused on the role of NECs as reviewers and approvers of research proposals and with a focus on their role in monitoring the process of research. The survey failed to enquire on the role played by NECs in ensuring dissemination of research findings to stakeholders and ensuring equitable risk-benefit-sharing of research with the communities involved.

Finally, the findings of this study suggest that NECs in the Region, by virtue of age, can be presumed to have attained an appreciable level of maturity and experience. The study does not provide an analysis to confirm operational independence and maturity of NECs, which can be drawn for example from the experiences of committee members, exposure to different genres of research proposals, studies of different disease types and communities, and complex vis-à-vis simple study designs. Such an analysis would provide a better understanding of NEC maturity beyond what can be inferred by virtue of old age.

### Ethics clearance

The WHO Regional Office for Africa Ethics Review Committee reviewed and provided approval for the study reported in this paper. The preamble to the questionnaire indicated that the aim of this survey was to gain a better understanding of the operations, as well as the legal and institutional frameworks supporting National Ethics Review Committees in African Countries. It also explained that the study would not have any direct benefits for individuals participating in the survey, and that the findings would be used for planning WHO support to countries for strengthening their NECs. The respondents were informed of their right to consent or not to consent to participating in the survey; and that there would be no consequences if one chose not to participate.

## Results and discussion

Thirty-three (33) out of a possible 41 countries (80.5 %) responded to the questionnaire. About 90 % of Anglophone countries responded compared to 71.4 % of Francophone countries. About 66.7 % respondents identified themselves as being members of NECs. Table 2 below summarizes the basic characteristics of NECs in responding countries highlighting findings on the existence of NECs and their respective dates of formation; countries in which NECs are created by legislation; the existence of law/policy establishing ethical standards for review of health research proposals; and those with secretarial and administrative support dedicated to NEC operations.

**Table 2:** Overview of basic characteristics of NECs in the African Region

Country	Existence and date of establishment	NEC established by law	Law/Policy guiding ethical standards	NEC has secretarial and administrative support
Algeria	Yes (1990)	Yes	Yes	Yes
Benin	Yes (2007)	Yes	Yes	Yes
Botswana	Yes (1984)	Yes	Yes	Yes
Burkina Faso	Yes (2002)	Yes	Yes	Yes
Burundi	Yes (2006)	No response	Yes	No
CAR	Yes (2005)	Nil	Nil	No
Chad	Nil	N/A	N/A	N/A
Congo	Yes (2007)	Nil	Nil	No
DRC	Yes (2003)	Nil	Nil	Yes
Eritrea	Yes (2006)	No response	Yes	No
Ethiopia	Yes (1995)	Yes	Yes	Yes
Equatorial Guinea	Nil	N/A	N/A	N/A
Gambia	Yes (1980)	Yes	Nil	Yes
Gabon	Yes (2007)	Nil	Nil	Yes
Ghana	Yes (2002)	Yes	Yes	Yes
Guinea	Yes (1999)	Yes	Yes	Yes
Kenya	Yes (2009)	Yes	No response	Yes
Lesotho	Yes (2007)	Yes	Nil	Yes
Liberia	Yes (2006)	Yes	Yes	Yes
Madagascar	Yes (1994)	Nil	Yes	Yes
Malawi	Yes	Yes	Yes	Yes
Mali	Yes (2002)	Yes	Yes	Yes
Niger	Yes (1999)	Yes	Yes	No
Nigeria	Yes (1980)	Yes	Yes	Yes
Senegal	Yes (2001)	Yes	Yes	Yes
Seychelles	Yes (1991)	(law enacted in 2012)	Yes	No response
Sierra Leone	Yes (1993)	Yes	Yes	Yes
South Africa	Yes (2006)	Yes	Yes	Yes
Swaziland	Yes (2006)	Yes	Yes	Yes
Tanzania	Yes (2002)	Yes	Yes	Yes
Uganda	Yes (1995)	Yes	Yes	Yes
Zambia	Yes (2008)	Yes	Yes	Yes
Zimbabwe	Yes (1974)	Yes	Yes	Yes

The survey shows that 31 of respondent countries (93.9 %) had NECs at the time of the survey. The pattern of establishment of NECs in the Region indicates three main waves. The first wave of early starters comprises of Zimbabwe whose NEC is the oldest in the Region and was created in 1974. The other early starters are The Gambia and Nigeria whose NECs were both created in 1980. The second wave comprises of NECs which were created between 1981 and 1999 and with an average age of 18.3 years. The NECs in the second wave include those of Algeria, Botswana, Ethiopia, Guinea, Madagascar, Niger, Seychelles, Sierra Leone, and Uganda. More than half of the NECs (54.5 %) were formed after the year 2000, which is the third wave coinciding with a number of significant global events such as the biotechnology revolution and the onset of the biopharmaceutical industry, the proliferation into the continent of HIV clinical trials and the human genome project. The NECs in the third wave have an average age of 8.9 years and include Benin, Burkina Faso, Burundi, Central African Republic, Congo, Democratic Republic of Congo, Eritrea, Gabon, Ghana, Kenya, Lesotho, Liberia, Mali, Senegal, South Africa, Swaziland, Tanzania, and Zambia.

These findings show that majority of countries in the Region have established NECs. Equatorial Guinea which at the time of the survey did not have a NEC established one in 2013. This leaves Chad as the only country, among those that participated in this study and according to the best of our knowledge, which does not have a NEC in place. It is also clear that NECs in the Region, except that of Equatorial Guinea, have been in existence for some time implying a certain level of maturity, accumulated experience, and operational independence [13].

- *Governance of health research ethics*

About 74.2 % of the responding countries had NECs established by either law or policy, and as expected all these countries except Kenya and Lesotho, indicated that they had guidance on the applicable ethical standards for reviewing health research proposals. A further analysis of the survey responses shows that most countries indicated that their NECs are established and governed by the application of policy instruments. These policies are most often instruments of interpreting provisions found in National Health Research or Public Health statutes. This could potentially create a legislative loophole on the authority and autonomy of NECs, which can be easily exploited to the detriment of the protection of research participants.

All respondent countries indicated that they referenced international guidelines for ethical research in their operations. The commonly cited international guidelines were the Helsinki Declaration, CIOMS guidelines [1, 8], WHO guidelines [10, 12], and the Belmont report [14]. All countries except Liberia, Niger and Nigeria indicated that all research institutions in their respective countries were required to adopt a policy regarding research ethics.

Functions, criteria for NEC membership, and composition: The question “*what are the main functions of the national ethics review committee*?” elicited a variety of responses. Only those responses commonly observed are reported here. They include ensuring ethical principles are observed in health research, including research on vulnerable groups and socially sensitive conditions such as HIV/AIDS; reviewing clinical trials; reviewing PhD study proposals; reviewing externally funded projects; approving agreements on biological sample and data transfer; providing policy advice to the ministry of health on health related research; conducting scientific review; undertaking regular capacity building for members; and monitoring ongoing approved studies. The Kenyan NEC was unique in that it does not review research proposals; its functions are to review national health research ethics guidelines, formulate policy, act as policy adviser to the ministry of health, arbitration and accreditation of institutional ERCs.

Some NECs in the Region perform the dual function of scientific and ethics review. This dual functionality is not surprising because scientifically unsound research is unethical. It could also be because of the overarching oversight function of NECs that these functions are vested under one entity. It is also noteworthy to observe that some NECs play the role of providing policy advice to the ministry of health. This can be potentially worrisome especially when questions of a NEC’s independence arise.

On the criteria of NEC membership, the study made an enquiry of how NECs were constituted by asking “are their written guidelines for constituting (i.e. selection of members) the national ethics review committee?” About 83.9 % NECs had written guidelines for selecting members. All NECs indicated that their composition was multidisciplinary in nature; however some skill sets such as those of non-physician health research professionals (namely statisticians, biomedical scientists and epidemiologists); physicians and lawyers were more represented than others. All NECs (100 %) had more than one member who was non-physician health research professional, 93.6 % of NECs had physicians, and 81.3 % had lawyers. About 56.3 % NECs had religious leaders and community representatives respectively. Only 34.4 % of the respondent NECs had members who were ethicists, pointing to a continued shortage of professionals formally trained in health research ethics in the Region. The gaps from the scarcity of these and other skills is possibly addressed by engaging experts or consultants from time to time. To this end it is instructive to note that about 76.7 % of the NECs indicated that they periodically engage consultants for technical support. In response to the question “*What in your opinion could be done to improve the performance of the national ethics committee*”, 26.7 % of NECS indicated that they needed to review policies guiding NEC membership to update the composition of the committee and make it inclusive of diverse skill-sets, and replace inactive members.

About 21.9 % NECs had representatives from patient advocacy groups, and 15.6 % had representatives from media. About 9.4 % NECs had members who were traditional practitioners, regulatory officers, representatives of professional associations respectively. This indicates a sub-optimal level of involvement of these constituencies.

b. *Existence of mechanisms for ethical review of health research*

All countries with NEC, except Ethiopia and Burundi, indicated that their NECs had written standard operating procedures (SOPs) for the conduct of business including clear guidelines for decision-making and managing scenarios of conflict of interest among Committee members. Those NECs with SOPs had established research proposal submission guidelines detailing required documents to be submitted with the research protocol. For most NECs the necessary accompanying documents included a brief summary of the research protocol, data collection instruments (case report forms and questionnaires), safety, pharmacological and toxicological data on the product being investigated, participant recruitment material and description of procedures for obtaining and documenting informed consent, principal investigator’s CV and documents of approval from other ethics review committees or regulatory agencies. Some NECs had additional requirements for example Nigeria required evidence of training in research ethics, Zambia required biological transfer agreements to be presented, and Gabon required evidence of insurance coverage for research participants. This finding further supports the assertion above linking average NEC ages to maturity, that NECs in the Region can indeed be assumed to have reached maturity and have established procedures for conducting ethical review. It must however be noted that maturity levels and experiences of NECs may differ from country to country.

Monitoring on-going research and penalties for non-compliance with ethical standards: About 76.7 % of respondent NECs reported to have mechanisms for monitoring on-going research. The most commonly applied mechanisms included a requirement for the submission of periodic written reports by the principle investigator (PI), unannounced audit/site visits by NEC representatives, and annual ethical review of previously approved projects. Incidentally, some NECs that had research monitoring mechanisms in place did not have a penalty system for non-compliance with relevant regulations and NEC decisions. Only 65.6 % of respondent NECs stated that they instituted penalties for non-compliance. The most common penalty was suspension of research (100 %); reporting to national regulatory authorities (71.4 %); and withdrawal of funds (19.1 %). Another less cited penalty was withdrawal of ethics approval renewal.

c. *Continuous ethics training and capacity requirements*

A number of capacity building initiatives [15, 16] for health research ethics have been put in place over time. However, there still remain capacity (both human and infrastructural) challenges in the Region. It was earlier stated that the Region faces a shortage of professionally trained ethicists and that NECS tend to have an over-representation of some skills. This, together with the rapidly evolving nature of health research due to scientific and technological advancements and emerging epidemics, calls for a concerted effort towards strengthening NECs capacities.

About 50 % of respondent NECs indicated that undergoing training was a requirement for committee members. About 66.7 % NECs indicated that capacity building opportunities were made available to committee members. This therefore means that there are some NECs who do not ensure that their members receive any training. Table 3 below summarizes the most commonly observed responses given to the question *“What in your view are the specific capacity needs of the national ethics committee?”*

**Table 3:** Capacity needs of NECs in the African Region

Capacity need	Observations (%)
Periodic training on international guidelines for health research ethics and bioethics generally	66.3 %
Training on evaluating clinical trials	53.3 %
Allocation of funds for the work of NEC (administrative and secretariat support)	50 %
Capacity for setting up and accrediting institutional ethics review committees including supporting them financially	16.7 %
Strengthening continuous review, oversight, auditing, monitoring and evaluation capacities	16.7 %
Database management – documentation and archiving of research protocols	13.3 %

Most NECs (66.3 %) indicated that they need periodic training on international guidelines for health research ethics. Periodic training provides an opportunity for committee members to update and refresh their knowledge on ethical values, concepts, and relevant international normative documents guiding health research ethics. Periodic training also allows NECs to stay abreast with contemporary ethical issues such as effective engagement with communities, risk and benefit analysis and determination, approached to obtaining voluntary informed consent, and equitable research benefits sharing. Periodic training becomes an important capacity strengthening avenue for NECs in the African Region where members have other full-time jobs and only serve on the NEC as an additional professional responsibility.

The second most common capacity need emerging from the survey, and indicated by 53.3 % of the NECS, was training on evaluating clinical trials. This comes as no surprise since the Region is experiencing an influx of clinical trials, particularly vaccine trials. About 16.7 % of the NECs indicated that they needed capacity for accrediting and financing institutional ethics review committees. This can be used as a good proxy indicator for maturity. Those NECs, such as Tanzania and Uganda, desirous of developing an accreditation framework and criteria for institutional ethics review committees can be construed to have attained a high level of functional and capacity maturity and are ready for the next phase of building other ERC’s capacities. About 16.7 % NECS indicated that they needed to strengthen their capacities for continuous review, oversight, auditing, monitoring and evaluation. About 13.3 % needed to capacities to improve their database management (filing and archiving) systems.

About 83.3 % NECs indicated that they had secretarial and administrative support. However, 50 % of the NECs indicated that they needed funds specifically allocated for the operations of the committee. The most commonly cited areas where funds would be used included enhancing health research monitoring activities, providing opportunities for members to attend international conferences and seminars, providing cash incentives for NEC members, conducting public awareness and community sensitization activities, and subscription to bioethics journals. This indicates that most NECs in the Region are not adequately funded.

### Suggestions for further research

This study provides a broad overview of the readiness of ethics review systems for a changing public health landscape in the WHO African Region. It provides basic information and data, which can be further interrogated through research, on the status of NECs as assessed against three inter-dependent themes namely, governance of health research; existence of mechanisms for ethical review of health research; and continuous ethics training and capacity requirements. Research to confirm anecdotal observations pointing to operational independence and maturity of NECs is one area that could be further explored.

Another possible strand for future policy research would be to identify factors, interests and mechanisms that will galvanize all health research stakeholders in the Region including governments and medical research councils; schools of public health and medicine; regional and national public health associations; the African Union, United Nations’ agencies and intergovernmental organizations; and industry to support formation and strengthening of NECs in the Region.

## Conclusions

The findings of this survey lead us to make a number of observations and policy recommendations. The finding on the presence of a possible legal lacuna for the operations of most NECs supports an argument that has been made before by Chima [1] on the need for the regional guidelines on health research drawn by a body such as the African Union, similar to the EU directives [17]. The guidelines would provide guidance on a number of issues, such as defining the standard of care; the use of placebo-controlled trials; process of obtaining voluntary informed consent in a community context; and community engagement. Such guidelines would take into account regional contextual specificities, and would be adapted by countries of the region into legislation.

It is clear that different NECs are at different stages of maturity, with some having a broader experience-base to draw from and others much less. While continued efforts by countries must be made to further strengthen human and infrastructural capacities, the fast evolving global public health landscape, such as testing investigational products for the Ebola virus disease would warrant proposing the creation of a regional platform/network, whose membership would be open to all NECs of the Region, to offer an opportunity for expeditiously and effectively addressing the challenge posed by weak and in some cases non-existent human resource capacities. Such a regional entity would serve to provide a forum for collaboration, networking and information sharing among NECs, would encourage harmonization of health research ethics policies and practices and procedures in countries of the Region, and would facilitate joint reviews especially when countries receive similar research proposals from one research group.

## Abbreviations

CIOMS: Council for International Organization of Medical Sciences
ERC: Ethics Review Committees
NEC: national ethics review committee
R & D: research and development
SOPs: standard operating procedures
WHO: World Health Organization
